# Is hypothyroidism rare in multidrug resistance tuberculosis patients on treatment? A systematic review and meta-analysis

**DOI:** 10.1371/journal.pone.0218487

**Published:** 2019-06-18

**Authors:** Habteyes Hailu Tola, Kourosh Holakouie-Naieni, Tadesse Lejisa, Mohammad Ali Mansournia, Mehdi Yaseri, Ephrem Tesfaye, Million Mola

**Affiliations:** 1 Tehran University of Medical Sciences-International Campus, School of Public Health, Department of Epidemiology and Biostatistics, Tehran, Iran; 2 Ethiopian Public Health Institute, Tuberculosis/HIV Research Directorate, Addis Ababa, Ethiopia; 3 St. Peter's Specialized Hospital, Research and Evidence Generation Directorate, Addis Ababa, Ethiopia; Jamia Hamdard, INDIA

## Abstract

**Background:**

Hypothyroidism is one of the adverse drug reactions that associated with Multidrug Resistant Tuberculosis (MDR-TB) medications. Extremely variable magnitude of hypothyroidism in MDR-TB patients has been reported from different parts of the world. However, there is no evidence that tried to estimate the pooled prevalence of hypothyroidism to confirm the rareness of hypothyroidism in MDR-TB patients on treatment. Therefore, we did a systematic review and meta-analysis to estimate the prevalence of hypothyroidism in MDR-TB patients on treatment, and to summarize the demographic and clinical characteristics of the patients.

**Methods:**

We conducted a systematic review and meta-analysis on studies reported around the world on the prevalence of hypothyroidism in MDR-TB patients on treatment. We searched electronic databases: PubMed/Medline, EMBASE, CINAHL, Science Direct, Academic Search Complete and Google scholar for English language articles without limiting publication year. We also reviewed the bibliographies of relevant studies and conducted an electronic search for relevant conference abstracts. Eligible studies were cross-sectional and cohort studies that included at least five participants. We used a random-effects model to estimate the pooled prevalence of hypothyroidism. The registration number of this review study protocol is CRD42018109237.

**Results:**

We included 30 studies and pooled data on a total of 6,241 MDR-TB patients. The crude prevalence of hypothyroidism was extremely heterogeneous. The pooled prevalence of hypothyroidism in MDR-TB patients on treatment was 17.0% (95% CI: 13.0–20.0). Ethionamide and para-aminosalicylic acid (PAS) were the most frequently reported drugs that associated with the occurrence of hypothyroidism.

**Conclusion:**

This review revealed that hypothyroidism is not a rare adverse drug reaction in MDR-TB patients on treatment. Ethionamide and PAS were the most frequently reported drugs that associated with the occurrence of hypothyroidism. Screening of hypothyroidism in MDR-TB patients on treatment is important while targeting patients on Ethionamide and PAS based treatment regimen.

## Background

Multidrug Resistance (MDR) and Extensively Drug Resistance (XDR) Tuberculosis (TB) are the most threat to human health across the world. MDR-TB is defined as a *Mycobacterium tuberculosis* strain resistance to the two powerful anti-tuberculosis isoniazid and rifampicin [1,2]. XDR-TB on the other hand refers to *Mycobacterium tuberculosis* strain resistance to isoniazid and rifampicin, any of the fluoroquinolones and at least one of the three injectable second-line drugs (amikacin, capreomycin or kanamycin) [1,2]. In 2017 alone, an estimated 3.5% of new cases and 18% of previously treated cases of MDR-TB have occurred globally [3]. The control of MDR-TB is much more difficult than drug-susceptible TB [4–7] which leads to further spread of resistant strains of the bacilli in several populations.

One of the most challenges in MDR-TB treatment is the severe adverse drug reactions associated with its medications [5,8–10]. The medication used for the treatment of MDR-TB is also considered as the most complicated because of its long duration, toxicity and economic burden it imposes [5,8–10]. In addition, MDR-TB drugs are known to cause multiple and severe adverse drug reactions that affect treatment outcome and quality of life of the patients [11–13]. Adverse drug reactions of MDR-TB drugs also leads to treatment interruption [14], which is the most important determinant of poor treatment outcomes such as prolonged morbidity, extensively or complete drug-resistance development, treatment failure and mortality [15]. The most frequently reported adverse drug reactions associated with MDR-TB drugs are skin rash, gastrointestinal symptoms, ototoxicity, electrolyte derangement, hepatotoxicity, nephrotoxicity, arthralgia, psychosis, suicidal tendencies, depression and hypothyroidism [1,2,12,16].

Hypothyroidism is one of the adverse drug reactions that cause life threatening side effect related to MDR-TB drugs [11,17,18]. The most likely MDR-TB drugs to cause hypothyroidism are Ethionamide (Eto), Thioamides (TA), Prothionamide (Pto) and Para-aminosalicylic acid (PAS) [11,19,20]. Although the effect of MDR-TB drugs on thyroid function is well cited in the literature [21–24], little is known about the exact mechanism through which these drugs influence thyroid function. Hypothyroidism is an uncommon adverse drug reaction which has vague and non-specific symptoms that can be easily missed by physicians [25]. Patients that developed hypothyroidism could manifest several symptoms which includes slow growth, puffy face, lethargy, hair loss, constipation, dry skin, enlarged thyroid gland, increased cholesterol level, irregular uterine bleeding, irritability, sensitivity to cold, sexual dysfunction, slow heart rate, weight gain, muscle weakness and stiffness or tenderness [26]. For these reasons, the World Health Organization (WHO) recommends screening of hypothyroidism at least per three or six months in MDR-TB patients on treatment [27]. However, laboratory supplies and techniques used to conduct thyroid function test are relatively rare and expensive than ordinary routine laboratory diagnosis [28,29]. In addition, access to quality assured laboratory diagnosis for such advanced test is a serious problem in low-income countries where the burden of MDR-TB is high [30,31]. Thus, screening and repeating the test per three or six months during the follow-up period could be impractical in low- income countries.

Available studies have reported an extremely variable prevalence of hypothyroidism among MDR-TB patients on treatment and have recommended screening of hypothyroidism during follow-up period [32–61]. However, there is no review that attempt to summarize the available literature to identify evidence that support recommendation on the screening of hypothyroidism in MDR-TB patients on treatment. Therefore, we did a systematic review and meta-analysis to estimate the pooled prevalence of hypothyroidism in MDR-TB patients on treatment, and to summarize the demographic and clinical characteristics of the patients.

## Methods

### Search strategy

We conducted a systematic review and meta-analysis of published articles to estimate the pooled prevalence of hypothyroidism and to summarize the demographic and clinical characteristics of the patients in MDR-TB patients on treatment following PRISMA standards (Preferred Reporting Items for Systematic Reviews and Meta-Analyses) ([62] and S1 Checklist). We systematically searched electronic databases: PubMed/Medline, Excerpta Medica Data Base from Elsevier (EMBASE), Cumulative Index to Nursing and Allied Health (CINAHL), Science Direct, Academic Search Complete and Google scholar for English language articles without limiting publication year. The electronic databases search was conducted from September 20, 2018 to November 10, 2018. We also reviewed the bibliographies of relevant studies, and conducted electronic search for conference abstracts. We used a search strategy by combining a key terms: “hypothyroidism”, “thyroid disorder”, “thyroid function”, “symptomatic hypothyroidism”, “drug-induced hypothyroidism”, “goitrous hypothyroidism”, “abnormal thyroid function”, “adverse drug reaction”, “drug side effect”, “tuberculosis”, “drug resistance”, “multidrug resistance”, “MDR-TB”, “Rifampicin resistance” and “treatment” both in Medical Subject Heading (MeSH) and free text terms. When necessary, we communicated with the included studies’ authors for clarification and additional information. Two authors (HHT and TL) independently reviewed the titles, abstracts and full articles of retrieved studies.

### Study inclusion and exclusion criteria

**Fig 1:**
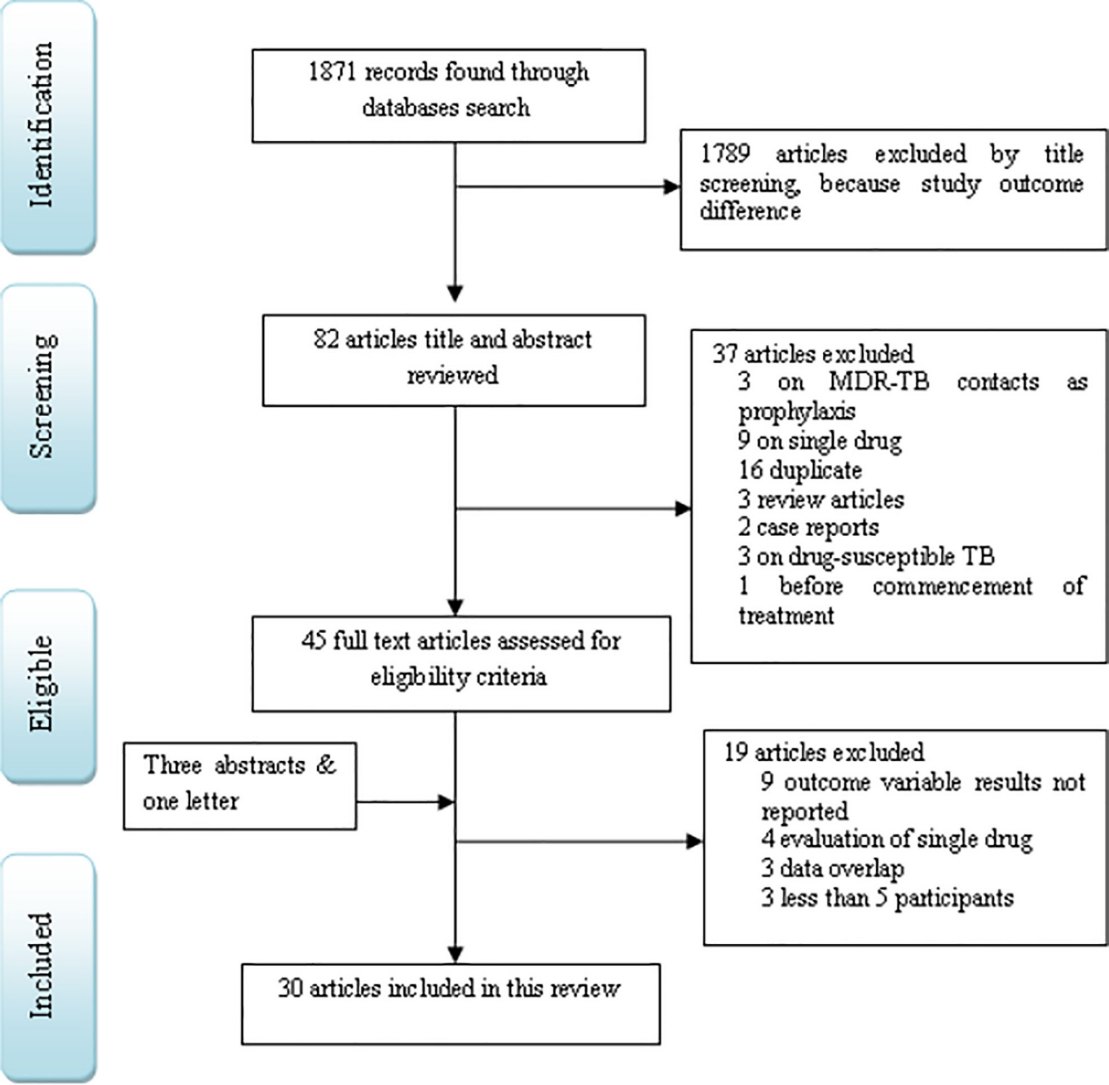
Study selection process flowchart.

We included a cohort studies that reported the prevalence of hypothyroidism at least on five MDR-TB patients on treatment and conducted in different parts of the world. We excluded studies that were conducted on single drug, latent TB treatment, on drug-susceptible TB and results not reported on key variables. We also excluded studies that were conducted before starting MDR-TB treatment, MDR-TB contacts as prophylaxis and data overlap (Fig 1 or S1 Diagram).

### Study quality assessment

We assessed the quality of the included studies by modified version of the Newcastle–Ottawa Scale (NOS) [63]. The scale assesses three key points (domains) of a given study: selection of participants; comparability of the groups; and outcome(s) assessment. We assigned stars for each point of the scale to categorize the studies into good, fair and poor quality based on the NOS [63]. A good quality study was scored 3 or 4 stars in participant selection, 1 or 2 stars in comparability of groups, and 2 or 3 stars in outcome(s) assessment. A fair quality study was scored 2 stars in participant selection, 1 or 2 stars in comparability of the groups, and 2 or 3 stars in outcome(s) assessment. A poor quality study was scored 0 or 1 star in participant selection, 0 stars in comparability of the groups and 0 or 1 star in outcome(s) assessment. In case of disparity between the two authors (HHT and TL) during study selection process, the disparities were resolved by the decision of the second author (KH).

### Data extraction

We extracted data from the included studies in to two databases separately. Our primary outcome was hypothyroidism that is measured by Thyroid Stimulated Hormone (TSH). Hypothyroidism is defined as abnormally below normal level of thyroid hormones in blood circulation [21]. In this review, we considered the presence of hypothyroidism if the authors of the included studies reported the prevalence in their results. We extracted information on characteristics of participants such as age range, mean of age, sex, HIV sero-status, treatment regimen, TB type (pulmonary versus extra pulmonary). We also extracted study’s characteristics such as first authors, publication year, study year, study setting, study duration, study design, study population, countries where the study was conducted, sample size and prevalence of hypothyroidism.

### Statistical analysis

We estimated the pooled prevalence of hypothyroidism with its 95% Confidence Interval (CI) using random effects meta-analysis model assuming the true effect size varies between studies [64]. We expressed the pooled prevalence of hypothyroidism as the ratio of numbers of patients that developed hypothyroidism to the total sample size, and the data was presented on forest plot. We also assessed the effect of sex on the thyroid hormone and estimated the pooled odds ratio with its 95% CI using random effect models. We assessed the heterogeneity in the prevalence of the different studies using Chi-square based Q test with significant level of p-value < 0.1 and I^2^ statistic with values above 75% as significant heterogeneity [65]. We also assessed potential publication bias with both funnel plot and Egger's test (p-value < 0.1 as significant level). In addition, we assessed the effects of the potential source of heterogeneity in the prevalence of hypothyroidism by subgroup analysis and moment based meta-regression models. We qualitatively summarized the drugs associated with hypothyroidism and carried out all data analysis by STATA version 14.

### Ethical consideration

Ethical clearance was not sought, as this review was based on previously published articles. However, the protocol of this study was pre-registered on PROSPERO (International prospective register of systematic reviews) University of York, Centre for Reviews and Dissemination with registration number CRD42018109237.

## Results

### Studies characteristics

We included 26 full articles, one letter to the editor [32] and three abstracts [33–35] that provided prevalence of hypothyroidism in MDR-TB patients on treatment [Fig 1, Table 1]. Studies included in this review were reported from 19 countries which cover three continents (Africa, Asia and Europe) [Table 1]. Seventeen studies were reported from Asian [32,34–40,42–44,46,48,53,54,60,61], eight from African [41,45,47,49,50,55,57,58] and five from European [33,48,52,56,59] countries. We categorized Russia, Turkey and Ukraine under European countries category. The majority of studies were reported from India (ten studies) [32,34–37,43,46,51,53,54] followed by South Africa (two studies) [41,47] and Egypt (two studies) [57,58].

**Table 1:** Characteristics of included studies.

First author, (publication year)	Study duration	Study setting	Study location	Study design	Sample size	Hypothyroidism n (%)	Hypothyroidism definition
Akshata et al (2015) [36]	2011–2014	Health facility	India	Retrospective cohort	484	19(3.9)	TSH value > 10 microIU/ml
Andries et al (2013) [37]	2006–2013	Health facility	India	Prospective cohort	69	37(54.0)	TSH value > 10 mIU/L after 3 months of treatment
Baghaei et al (2011) [38]	2006–2009	Health facility	Iran	Retrospective cohort	80	1(1.3)	——-
Bares et al (2016) [39]	2011–2012	Health facility	Pakistan	Prospective cohort	50	39(78.0)	Adverse effect of certain anti-tuberculosis drugs
Bhatt et al (2017) [40]	Jul-Nov, 2012	Health facility	Nepal	Prospective cohort	101	6(6.4)	——-
Brust et al (2013) [41]	2008–2011	Health facility	South Africa	Retrospective cohort	73	26(36.0)	TSH level >8 mIU/L
Cheung et al (2018) [42]	1999–2017	Health facility	Australia	Retrospective cohort	29	9(31.0)	TSH above upper limit
Chhabra et al (2011) [32]	2005–2011	Health facility	India	Prospective cohort	54	6(11.0)	——-
^a^Gupta et al (2011) [33]	——-	Health facility	United Kingdom	Prospective cohort	5	4(80.0)	——-
Hire et al (2014) [43]	Jan-Dec, 2012	Health facility	India	Prospective cohort	110	1(0.9)	
Hoa et al (2015) [44]	2010–2012	Health facility	Vietnam	Prospective cohort	282	3(1.3)	——-
Huerga et at (2017) [45]	2006–2012	Health facility	Kenya	Retrospective cohort	169	31(18.0)	TSH > 10 mU/l
Isaakidis et al (2012) [46]	2007–2011	Community	India	Prospective cohort	67	21(31.0)	TSH > 10mIU/L
Jacobs et al (2014) [47]	2010–2011	Health facility	South Africa	Retrospective cohort	350	29(8.3)	——-
^a^Kala et al (2008) [34]		Health facility	India	Retrospective cohort	110	12(12.9)	——-
Matveyeva et al (2017) [48]	——-	Health facility	Ukraine	——-	30	5(16.7)	Increased TSH value and decline in T4 levels
Meressa et al (2015) [49]	2009–2014	Health facility	Ethiopia	Retrospective cohort	612	105(17.2)	——-
Modongo et al (2012) [50]	1-30/Jan/2007	Community	Botswana	Prospective cohort	213	73(34.3)	TSH > 10.0 μIU/l
Munivenkatappa et al (2016) [51]	2014–2015	Community	India	Prospective cohort	188	43(23.0)	TSH value ≥10 mIU/ml
Nathanson et al (2004) [52]	1998–2002	Community	Estonia, Latvia	Retrospective cohort	818	29(3.5)	TSH > 10 mU/L
Prasad et al (2016) [53]	2009–2010	Health facility	India	Prospective cohort	98	1(0.8)	TSH > 10.0 IU/ml
^a^Prasad et al.(2013) [35]	2009–2012	Health facility	India	Prospective cohort	98	3(3.1)	——-
Saharia et al (2015) [54]	2012–2013	Health facility	India	Prospective cohort	99	5(5.1)	TSH >10 mIU/mL
Satti et al (2012) [55]	2007–2009	Community	Lesotho	Retrospective cohort	186	129(69.0)	TSH value > 10.0 mIU
Shin et al (2007) [56]	2000–2002	Community	Russia	Retrospective cohort	244	42(17.2)	TSH >10.0 IU/ml
Tag El Din et al (2015) [57]	2009–2012	Health facility	Egypt	Retrospective cohort	107	11(10.3)	——
Tag El-Din et al (2015) [58]	2006–2009	Health facility	Egypt	Retrospective cohort	138	13(9.4)	TSH value >10 mU/L
Törün et al (2005) [59]	1992–2004	Health facility	Turkey	Retrospective cohort	263	3(1.1)	TSH > 10 mU/L
Yang et al (2017) [60]	2006–2011	Health facility	South Korea	Retrospective cohort	256	6(2.3)	TSH > 10.0 IU/mL
Zhang et al (2017) [61]	2009–2016	Health facility	China	Ambispective cohort	751	148(19.7)	TSH > upper limit

———the information or value not reported, UK-United Kingdom, TSH-Thyroid Stimulating Hormone, IU- International Unit, a-abstract, l-letter

Twenty four studies were based on health facility (hospital, TB clinic, research center and MDR-TB treatment center), while six were community based [Table 1]. In terms of study design, 15 were retrospective cohort study, 13 prospective cohort study, one ambispective cohort study [61] and one study [48] has not reported the study design [Table 1].

**Fig 2:**
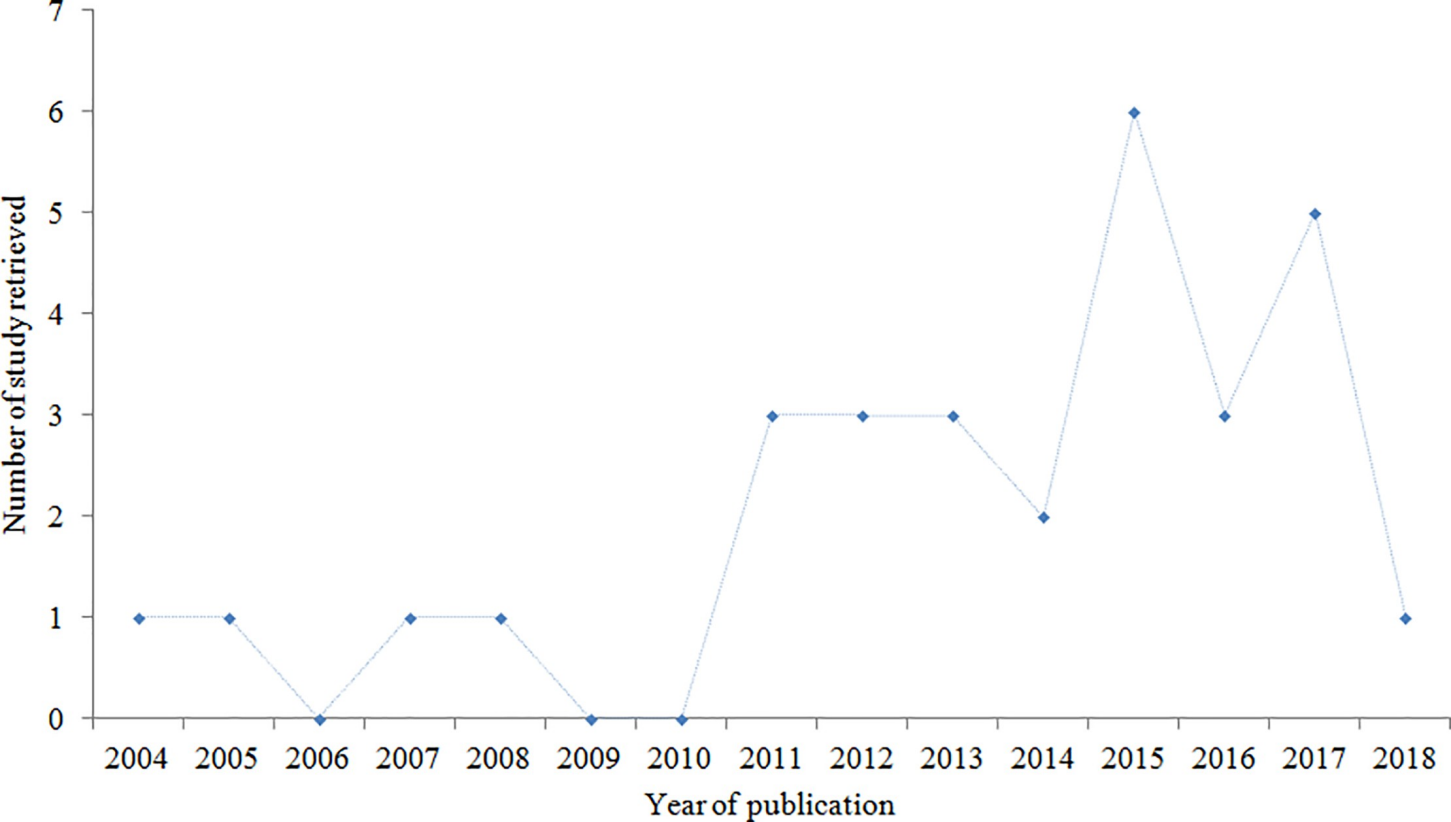
Number of studies retrieved over the publication years.

Fig 2 depicts the number of studies identified by year of publication. The publication year of the articles ranged from 2004 to 2018, and majority of the studies were published after 2010 [Fig 2, Table 1]. The study duration ranges from one month to 12 years [Table 1]. The minimum sample size used by the included studies was five participants [33] and the maximum 818 [52] [Table 1]. Twenty studies were clearly defined hypothyroidism, but eleven studies did not provide the definition of hypothyroidism [Table 1]. Majority of the studies used TSH concentration level to assess the presence of hypothyroidism [Table 1].

Six studies [32,36,37,39,51,54] used immunoassay based techniques as the method to measure TSH concentration, while 14 studies [38,41,42,44,46,47,49,55–61] retrieved the value of TSH concentration from record (treatment card or laboratory report). However, ten studies [33–35,40,43,45,48,50,52,53] did not report how TSH concentration was measured, and from where data on TSH concentration was collected.

Seventeen studies did not provide age ranges of their participants, and the remainder covered adult participants [Table 2]. In addition, eight studies did not provide either mean or median of age, but 22 studies provided either mean or median of age [Table 2]. Thirteen studies reported HIV sero-reactive status of the participants with the pooled prevalence of HIV co-infection with MDR-TB was 30.0% (95% CI: 21.0–40.0). Analysis from six studies shown that the pooled prevalence of pulmonary TB was 89.0% (95% CI: 84.0–93.0)[37,38,42,46,49,61], while the pooled prevalence of extra-pulmonary TB as estimated from the same six studies was 12.0% (95% CI: 7.0–17.0).

**Table 2:** Characteristics of study participants.

First author (publication year)	Age range (in year)	Mean age (in year)	Treatment regimen	Drug associated	Drugs used for MDR-TB treatment
Akshata et al (2015) [36]	14–55	31.7	Standardized	——	Levofloxacin, Ethionamide, Ethambutol, Cycloserine, PAS
Andries et al (2013) [37]	——	——	Individualized	Ethionamide and PAS	Pyrazinamide, Capreomycin, Ethionamide, Cycloserine, PAS
Baghaei et al.(2011) [38]	14–81	40.64	Standardized	——	Amikacin, Cycloserine, Prothionamide, Ofloxacin, Ethambutol, Pyrazinamide
Bares et al (2016) [39]	14–50	25.5	Individualized	——	Isoniazid, Rifampicin, ethambutol, pyrazinamide, streptomycin, PAS
Bhatt CP et al (2017) [40]	——	31.2	——	——	——
Brust et al (2013) [41]	——	34	Standardized	Ethionamide and PAS	Kanamycin, Ofloxacin, Cycloserine, Ethionamide, Pyrazinamide, Ethambutol
Cheung et al (2018) [42]	22–47	35	————	Prothionamide and PAS	Prothionamide and PAS
^l^Chhabra et al.(2011) [32]	——	38.57	——	——	
^a^Gupta et al (2011) [33]	29–40	——	——	Prothionamide and PAS	Regimens containing PAS and Prothionamide.
Hire et al (2014) [43]	18–79	——	——	Ethionamide	Kanamycin, Levofloxacin, Ethionamide,Pyrazinamide, Ethambutol, Cycloserine
Hoa et al.(2015) [44]	30–45	42.35	Standardized		Amikacin, Kanamycin, Ofloxacin, Ethionamide, Cycloserine, PAS, Ethambutol
Huerga et at.(2017) [45]	——	——	Standardized	——	Kanamycin, Capreomycin, Levofloxacin, Prothionamide, Cycloserine, PAS
Isaakidis et al (2012) [46]	——	——	Standardized	Ethionamide and PAS	Pyrazinamide, Isonized, Rifampicin, Ethionamide, Cycloserine and PAS
Jacobs et al.(2014) [47]	——	35.65	Standardized	Ethionamide	Kanamycin, Amikacin, Ofloxacin, Ethionamide, Terizidone, Pyrazinamide
^a^Kala et al (2008) [34]	——	48	Individualized	——	——
Matveyeva et al.(2017) [48]	——	38.57	Individualized	Ethionamide and PAS	Ethionamide and PAS
Meressa et al.(2015) [49]	——	——	——	——	Levofloxacin, Ethionamide, PAS, Pyrazinamide, Kanamycin, Cycloserine
Modongo et al (2012) [50]	28–48	37	——	Ethionamide and PAS	Ethionamide and PAS
Munivenkatappa et al (2016) [51]	——	——	——	Ethionamide and PAS	Ethionamide and PAS
Nathanson et al.(2004) [52]	——	——	Standardized	Thioamides and PAS	Amikacin, Capreomycin, Cycloserine, Ethionamide, Kanamycin, PAS
Prasad et al (2016) [53]	>18	29.3	Standardized	Ethionamide	Streptomycin, Rifampicin, Isoniazid, Ethambutol, Pyrazinamide
^a^Prasad et al.(2013) [35]	——	——	——	Ethionamide	Kanamycin, Cycloserine, Ethionamide, Pyrazinamide
Saharia et al.(2015) [54]	31352	32.29	Standardized	Ethionamide and PAS	Ethionamide and PAS
Satti et al.(2012) [55]	——	——	——	Ethionamide and PAS	PAS, Cycloserine, Pyrazinamide, Ethionamide, Prothionamide
Shin et al.(2007) [56]	17–65	31.8	Individualized	PAS	Ethionamide, PAS, Cycloserine, Capreomycin, Kanamycin, Streptomycin
Tag El Din et al (2015) [57]	15–67	37.1	Individualized	Kanamycin	Kanamycin, Ethionamide, Cycloserine, PAS, Ofloxacin, Amikacin
Tag El-Din et al (2015) [58]	15–67	37.6	——	Ethionamide and PAS	Ofloxacin, Ethionamide, Cycloserine, PAS, Kanamycin, Amikacin
Törün et al.(2005) [59]	14–68	37.8	Individualized	——	Isonized, Rifampicin, Ethambutol, Streptomycin, Prothionamide, Amikacin, Kanamycin
Yang et al (2017) [60]	——	42.1	Individualized	——	Isonized, Ethambutol, Streptomycin, Kanamycin, Ethionamide, PAS
Zhang Y et al (2017) [61]	——	——	Standardized	——	Pyrazinamide, Amikacin, Kanamycin, Capreomycin, Levofloxacin, PAS

PAS- para-aminosalicylic acid, a-abstract, l-letter

Twelve studies did not report the regimen used for the treatment of MDR-TB patients [Table 2]. However, 11 studies reported standardized regimen, while seven studies reported individualized regimen [Table 2]. The majority of studies (eighteen) reported the drugs that are associated with hypothyroidism [Table 2], including Ethionamide, PAS, Kanamycin, Thioamide and Prothionamide [Table 2]. Ethionamide and PAS were the most frequently reported drugs that are associated with hypothyroidism [Table 2]. Four studies [37,42,50,51] reported the proportion of hypothyroidism by sex, and we pooled the odds ratio of these four studies to assess the effect of sex on the thyroid hormone in MDR-TB patients on treatment. As a result, the pooled odds ratio between female and male was 0.534 (95% CI 0.262–1.09) by considering female as risk category.

### Pooled prevalence of hypothyroidism

We pooled data on 6,241 MDR-TB patients on treatment to estimate the pooled prevalence of hypothyroidism in this meta-analysis. We used random effects model because the results of overall Chi-square based Q test and I^2^ statistic (variation in effect sizes attributable to heterogeneity) shown high heterogeneity between the results of the studies (Q = 1154.00, df = 29, p-value < 0.001 and I^2^ = 97.49%) for hypothyroidism prevalence estimation.

**Fig 3:**
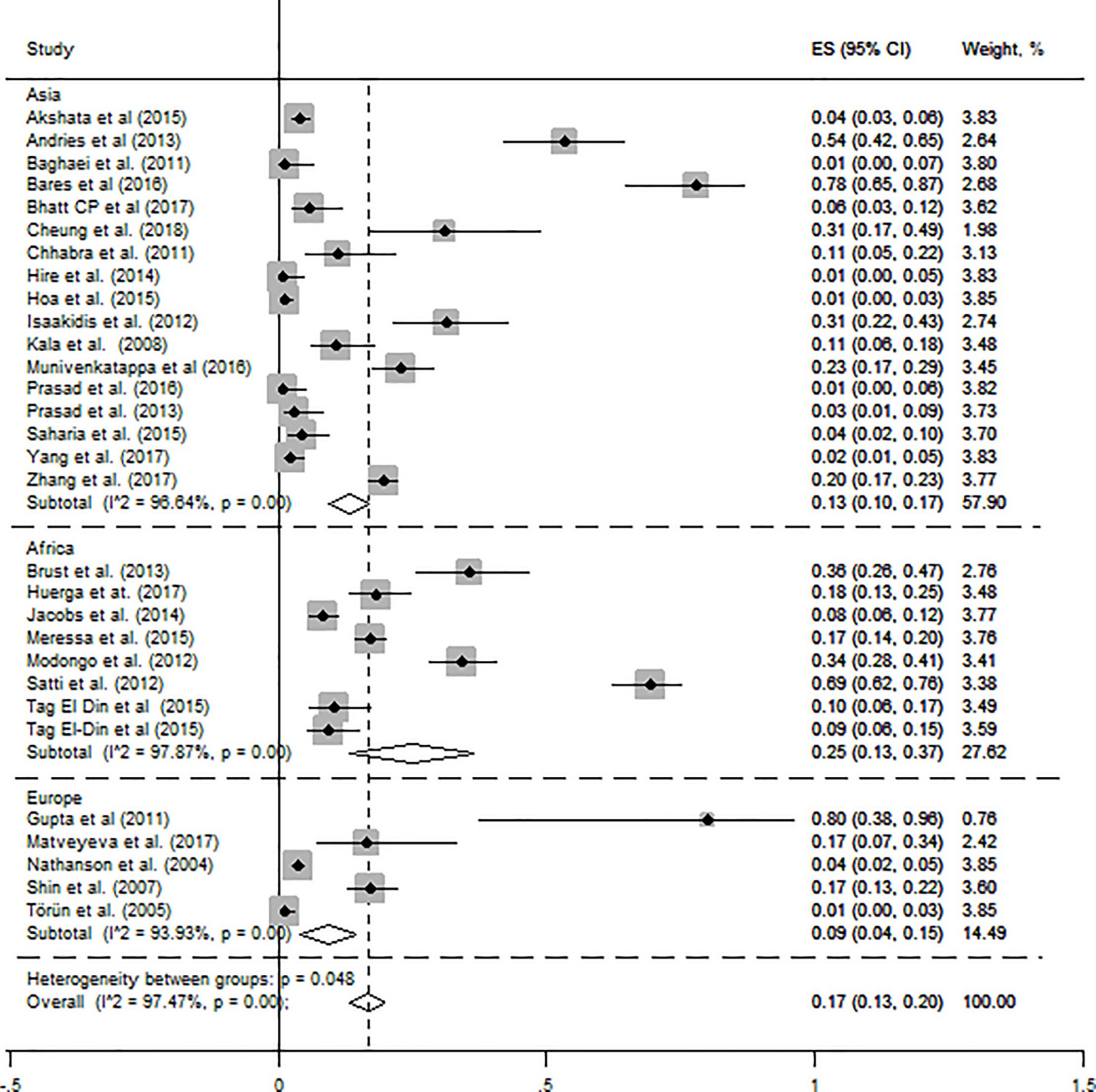
Pooled prevalence of hypothyroidism in MDR-TB patients on treatment with 95% CI (the estimate weighted based on random effects model): ES-Effect Size equivalent to prevalence, CI-Confidence interval.

Fig 3 shows a forest plot with effect size (ES-prevalence) and 95% confidence interval. The crude prevalence of hypothyroidism ranges from 1.0% to 80.0% [Fig 3]. The overall pooled hypothyroidism prevalence was 17.0% (95% CI: 13.0–20.0) [Fig 3]. The pooled prevalence of hypothyroidism in Africa was 25.0% (95% CI; 14.0–37.0) which was significantly higher than Asian prevalence of 13.0% (95% CI; 10.0–17.0), and European prevalence of 9.0% (95% CI; 4.0–15.0) [Fig 3].

### Subgroup analysis

Table 3 depicts subgroup analysis results based on study setting, study design, treatment regimen and continent from where the study was reported. The results of all subgroup analysis have shown significant heterogeneity between groups and within the group [Table 3].

**Table 3:** Subgroup analysis based on study setting, study design, regimen type and continent from where the study reported.

				Heterogeneity tests			
Group Variable		Number of study	Hypothyroidism prevalence, %,(95% CI)	*X*^*2*^	d.f.	I^2^	p-value
Study setting	Health facility	24	13.0(10.0–16.0)	612.50	23	96.24	<0.001
	Community	6	30.0(11.0–49.0)	496.84	5	98.99	<0.001
Study design	Retrospective cohort	15	15.0(10.0–19.0)	592.97	14	97.64	<0.001
	Prospective cohort	11	20.0(14.0–26.0)	437.59	12	97.26	<0.001
	Ambispective cohort	1	20.0(17.0–23.0)	—	0	—-	—
	Not reported	1	17.0(7.0–34.0)		0	—-	—
Regimen type	Standardized	11	12.0(8.0–17.0)	278.44	10	96.41	<0.001
	Individualized	7	14.0(8.0–20.0)	131.34	6	95.43	<0.001
	Not reported	12	25.0(16.0–33.0)	725.00	11	98.48	<0.001
Continent	Asia	16	13.0(10.0–17.0)	449.71	15	96.66	<0.001
	Africa	10	25.0(14.0–37.0)	600.68	9	98.50	<0.001
	Europe	5	9.0(4.0–15.0)	65.93	4	93.93	<0.001
Overall		31	16.0(13.0–20.0)	1155.43	30	97.40	<0.001

CI: Confidence Interval; d.f.: degree of freedom

### Meta-regression analysis

We assessed the effects of year of study and sample size of each study on heterogeneity between studies using meta-regression model [Table 4]. Sample size (p = 0.002) was significantly predicted prevalence of hypothyroidism heterogeneity across the studies [Table 4]. However, study year (p = 0.300) was not significantly predicted prevalence of hypothyroidism heterogeneity [Table 4].

**Table 4:** Meta-regression analysis for year of study and sample size as a reason of heterogeneity on the prevalence of f hypothyroidism.

	Unadjusted Model			Adjusted Model		
Predictive Variable	ß (95% CI)	SE	p-value	ß (95% CI)	SE	p-value
Year of study	0.69(-3.37–4.76)	1.98	0.729	1.86(-1.64–5.36)	1.70	0.286
Sample size	0.10(0.040–0.16)	0.03	0.002	0.105(0.044–0.17)	0.30	0.002

SE-Standard error, ß-regression coefficient, CI- 95% Confidence interval

### Publication bias

**Fig 4:**
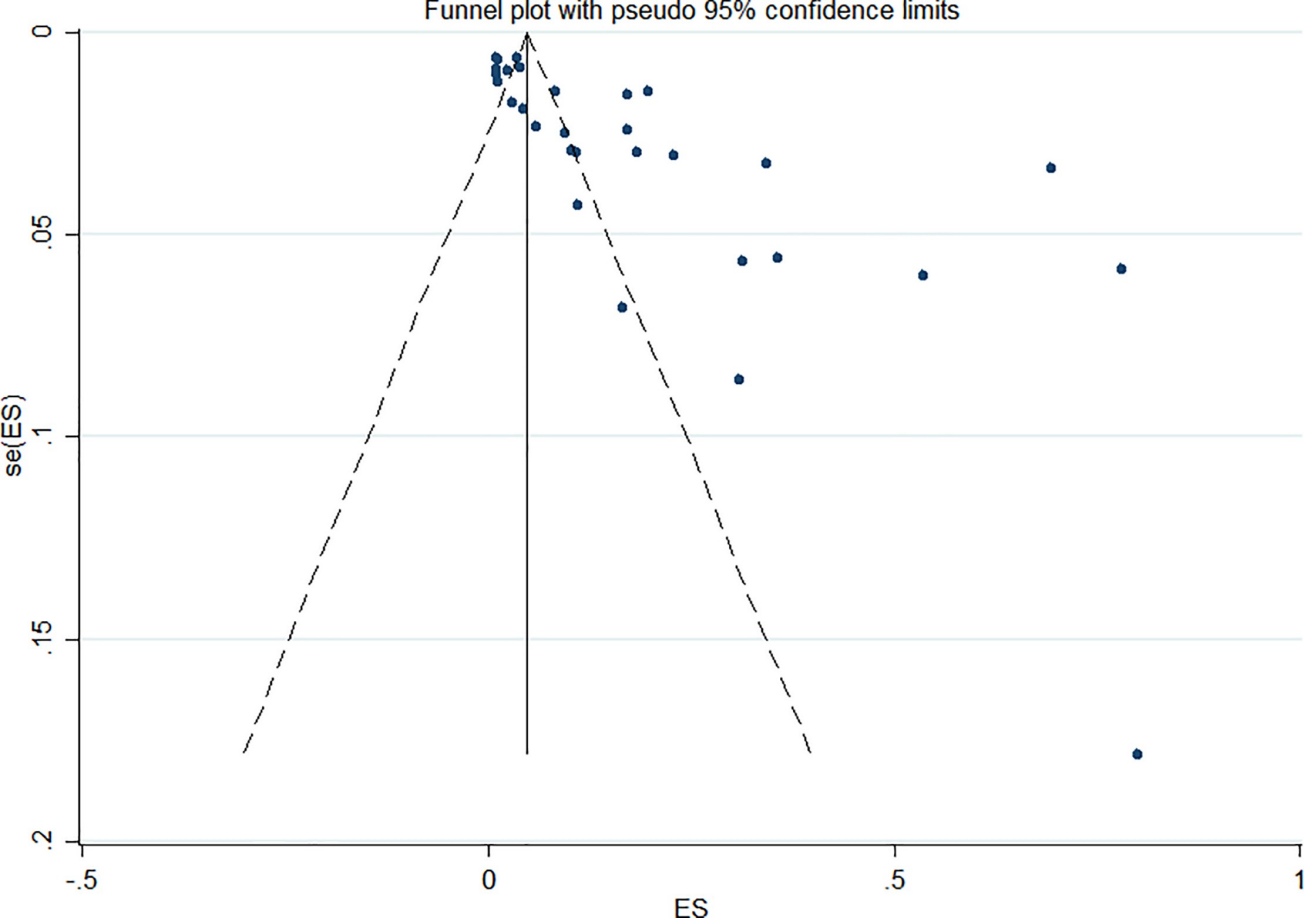
Funnel plot of the 30 estimates available for meta-analysis (SE- Standard error, ES–Effect size: Prevalence).

The funnel plot (Fig 4) was asymmetrical, which suggested the possibility of publication bias. An Egger’s test result (p = 0.01) was also confirmed the presence of publication bias in the included studies in estimated prevalence of hypothyroidism.

## Discussion

Medications used to treat MDR-TB are known to cause multiple and severe adverse drug reactions that affect treatment outcome, treatment adherence and patient's quality of life [11–13]. Hypothyroidism is one of adverse drug reactions that cause life threatening conditions in patients [11,17,18]. However, there is little evidence that attempted to summarize the available literatures to estimate the pooled prevalence, and summarize sociodemographic and clinical characteristics of patients. We included 30 studies reported from 19 countries across three continents (Africa, Asia and Europe), and pooled data on a total of 6,241 MDR-TB patients on treatment. The data reported from the included studies were mostly from small and heterogeneous single centre studies. The result of our meta-analysis revealed 17.0% of pooled prevalence of hypothyroidism in MDR-TB patients on treatment. Our review also suggested that hypothyroidism is not a rare adverse drug reaction in MDR-TB patients on treatment. The pooled prevalence of hypothyroidism in Africa (25.0%) was significantly higher than in Asia (13.0%) and Europe (9.0%). Furthermore, Ethionamide and PAS were the most frequently reported drugs that were associated with the occurrence of hypothyroidism.

The burden of hypothyroidism should not be considered as a rare adverse drug reaction in MDR-TB patients on treatment, because the pooled estimate of our review indicated high prevalence (17.0%) of the problem. This finding was consistent with the previous review report that pooled the results of three studies in which the pooled prevalence of hypothyroidism was 15.9% [17]. In contrast to our finding, a review reported by Wu et al [66] indicated low (3.6%) prevalence of hypothyroidism in MDR-TB patients on treatment. This difference might be due to differences in included studies or diagnosis upper limit used by the included studies. The treatment of MDR-TB is a difficult phenomenon that challenges patient management due to its long term treatment, expensive cost and severe adverse drug reactions [5,8–10]. The adverse drug reactions are more common and severe than the standard treatment for drug-susceptible TB, which reduce patient adherence and result to poor treatment outcomes [11,12,14,15]. Thus, it is critical for physicians to monitor the patient thyroid hormone status and provide appropriate treatment for patients parallel to the standard MDR-TB medication to support a successful treatment outcome.

There was a significant difference in the pooled prevalence of hypothyroidism within the group and between the groups during subgroup analysis. This might be due to variation in the prevalence of hypothyroidism at individual study level, and the heterogeneity due to potential confounding factors at the study level such as other drug adverse reactions and availability of hypothyroidism or chronic conditions before MDR-TB treatment started, which could aggravated by MDR-TB and its medications.

The pooled prevalence of hypothyroidism in African countries was significantly higher than in Asian and European. This probably due to the presence of autoimmune disease, infections [67], and food availability and diversity [68]. The difference in baseline thyroid hormone concentration level of patients in each study could also be the main reason of variation in the pooled prevalence of hypothyroidism between the groups and within the group.

Ethionamide and PAS were the most frequently reported drugs that were associated with hypothyroidism. As we did not obtain the adjusted measure of association from the included studies, we could not estimate the pooled measure of associations to know the effect size of these two drugs on the occurrence of hypothyroidism. More importantly, several studies did not report the diagnostic criteria of hypothyroidism which could help in the estimation of the burden of hypothyroidism and the level of association of these drugs. For instance 11 studies did not report the definition of hypothyroidism which might be the potential source of heterogeneity on the estimated prevalence. All these conditions may have an impact on the accuracy of the results. However, the finding of this review highlights the need of standardized data registration and reporting forms in MDR-TB programs, which could have important implications on the international MDR-TB control program. Thus, based on this finding physicians who treat MDR-TB patients should be aware of the occurrence of hypothyroidism during the treatment of MDR-TB. In addition, our pooled estimated prevalence of hypothyroidism, summarized demographic, and clinical characteristics could help as a point of reference for the physicians to take evidence-based actions carefully in patient monitoring and management.

In this review, the pooled odds ratio shows female sex is not the risk of hypothyroidism in MDR-TB patients on the treatment. Although, we could not find previous review study that pooled the effect of female sex on the thyroid hormone, available primary studies shows contradicted results with our review finding in which female sex was not the risk of hypothyroidism in MDR-TB patients on treatment [69,70]. This contradiction occurred most probably due to study population difference and treatment status difference. In this review all participants were on MDR-TB treatment, but previous primary studies’ populations were not on MDR-TB treatment. In addition, this difference might occur due to small amount of studies included to pool the odds ratio in this review which might under estimate the effect of female sex on the occurrence of hypothyroidism.

A major strength of this review is the inclusion of a large number of studies and the use of large sample size, which made the estimation of pooled prevalence of hypothyroidism more precise. In addition, the large sample size further facilitated rigorous subgroup analyses to investigate the potential sources of heterogeneity on the estimation of hypothyroidism prevalence. Moreover, we employed a random effects model to address heterogeneity among the studies.

The main limitation of this review was inclusion of studies published in English language only. This might induce publication bias. In addition, although individual data pooling is very important to perform subgroup analysis, statistical interaction test and refine dose-response curve, we did not use individual data pooling in this analysis. This might limited our sub-group analysis to further explore the source of heterogeneity. Moreover, there was inconsistent reporting of important participants’ characters such as prevalence of hypothyroidism by TB type (pulmonary versus extra pulmonary), HIV sero-status, antiretroviral treatment status, treatment regimen, treatment duration and baseline TSH concentration level. These inconsistences limited our analysis to further determine the potential source of heterogeneity on the prevalence of hypothyroidism. Particularly, the absence of baseline hypothyroidism status might induce estimation bias to the pooled prevalence of hypothyroidism. Thus, measuring and accounting for baseline hypothyroidism is very important to estimate the independent prevalence of hypothyroidism due to MDR-TB drugs. Moreover, few studies reported the drugs and other associated factors on the hypothyroidism. This limited our analysis to estimate the pooled effect size of drugs and other factors that are associated with hypothyroidism, while accounting for the potential confounders.

## Conclusion

Our review indicated that hypothyroidism is not a rare drug adverse reaction in MDR-TB patients on treatment. Ethionamide and PAS were the most frequently reported drugs that were associated with the occurrence of hypothyroidism. Screening hypothyroidism among MDR-TB patients on treatment is vital, particularly targeting patients on regimen containing Ethionamide and/or PAS to treat the condition at early stage. In addition, larger scale prospective study is so important to further explore the effect of MDR-TB drugs on thyroid hormone function while controlling baseline hypothyroidism and other confounding factors.

## Supporting information

S1 Checklist (DOC)

S1 Diagram (DOC)

S1 Dataset (XLSX)
