# Supplementary material for: Is hypothyroidism rare in multidrug resistance tuberculosis patients on treatment? A systematic review and meta-analysis
Source: PLoS One. 2019 Jun 18;14(6):e0218487. doi: 10.1371/journal.pone.0218487 (PMC6581430; doi:10.1371/journal.pone.0218487)
Supplement: S1 Diagram — (DOC) [file pone.0218487.s002.doc]

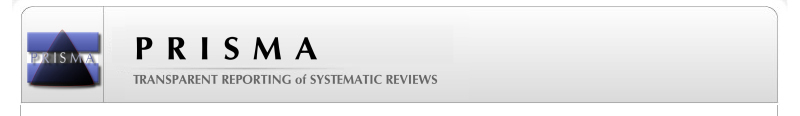
**PRISMA 2009 Flow Diagram**

1789 studies excluded by title screening, because study outcome difference

1871 records found through databases search

37 studies excluded

3 on MDR-TB contacts as prophylaxis

9 on single drug

16 duplicate

3 review articles

2 case reports

3 on drug-susceptible TB

1 before commencement of treatment

82 studies title and abstract reviewed

45 full text studies assessed for eligibility criteria

19 studies excluded

9 outcome variable results not reported

4 evaluation of single drug

3 data overlap

3 less than 5 participants

30 articles included in this review

Identification

Screening

Eligible

Included

Three abstracts & one letter
